# P33 Investigation to assess the impact of treatment-dose co-trimoxazole on renal function and serum potassium

**DOI:** 10.1093/jacamr/dlaf118.040

**Published:** 2025-07-14

**Authors:** Matthew Youngman, Nicholas Jones, William Petchey

**Affiliations:** West Suffolk Hospital, Bury St Edmunds, UK; West Suffolk Hospital, Bury St Edmunds, UK; West Suffolk Hospital, Bury St Edmunds, UK

## Abstract

**Background:**

As a WHO ‘Access’ group antibiotic with broad-spectrum antimicrobial activity, co-trimoxazole is an attractive alternative to ‘Watch’ and ‘Reserve’ group treatments for many patients. With favourable pharmacokinetic properties, it is ideally suited for treating patients on complex outpatient antimicrobial therapy (COpAT) pathways. However, acceptability of co-trimoxazole can be limited by clinician concerns over the potential for nephrotoxicity. We sought to investigate the prevalence of acute kidney injury (AKI) and hyperkalaemia in inpatients receiving standard-dose and high-dose co-trimoxazole at our centre.

**Methods:**

This was a single-centre retrospective study of all patients aged ≥18 years prescribed treatment-dose co-trimoxazole over a 3-year period at West Suffolk Hospital (WSH). Data was extracted from e-Care (Cerner) and Metavision to ascertain the presence of acute kidney injury (AKI) or hyperkalaemia during their inpatient admission, followed by manual review of patient notes to identify time taken to recovery and additional contributing factors. ‘High-dose’ was defined as 30-120mg/kg dosing based on renal function. Patients identified with AKI and hyperkalaemia were classified using Kidney Disease Improving Global Outcomes (KDIGO), NG148 and laboratory criteria.

**Results:**

589 WSH inpatients received treatment-dose co-trimoxazole during the study period; 21/589 (3.6%) at high-dose and 568/589 (96.4%) at standard-dose. AKI was observed in 55/568 (9.6%) of the standard-dose group, which equates to a 1000-fold greater likelihood of AKI in this cohort than the <1/10 000 suggested by the summary of product characteristics (SmPC). Risk of AKI was higher in the high-dose group, with 6/21 (28.6%) affected. There were no observed cases of AKI with severity >stage 1 in the standard-dose group, whereas stage 2 AKI was seen in 1/21 (4.7%) and none with stage 3 in the high-dose group. None of the AKI patients in the study required renal replacement therapy. 90 day recovery of AKI was observed in 52/55 (95%) of the standard-dose group and 3/6 (50%) of the high-dose group. 2/21 (9.5%) died with gradually deteriorating renal function prior to 90 days post-treatment, both of which were in the high-dose group. Neither death was attributed to renal failure. Hyperkalaemia with serum potassium >5.4 mmol/L was observed in 15/568 (2.6%) of the standard-dose group, which is 4-fold lower than the 10% risk suggested by the SmPC. Furthermore, only 12/568 (2.1%) had serum potassium >6.0 mmol/L, which is the local threshold for recommended medical management of hyperkalaemia. Hyperkalaemia risk was greater in the high-dose group, with 4/21 (19%) having potassium >5.4 mmol/L and 2/21 (9.5%) >6.0 mmol/L. 424/589 (72%) of hyperkalaemia was found to be present in the context of an acute deterioration in creatinine clearance.

**Conclusions:**

AKI was significantly more common in our cohort than is suggested by the SmPC. However, it is generally mild and transient at standard-dosing, meaning risk can be mitigated by appropriate toxicity monitoring.

